# Sequencer-Based Capillary Gel Electrophoresis (SCGE) Targeting the rDNA Internal Transcribed Spacer (ITS) Regions for Accurate Identification of Clinically Important Yeast Species

**DOI:** 10.1371/journal.pone.0154385

**Published:** 2016-04-22

**Authors:** Xin Hou, Meng Xiao, Sharon C.-A. Chen, He Wang, Li Zhang, Xin Fan, Zhi-Peng Xu, Jing-Wei Cheng, Fanrong Kong, Yu-Pei Zhao, Ying-Chun Xu

**Affiliations:** 1 Department of Clinical Laboratory, Peking Union Medical College Hospital, Chinese Academy of Medical Sciences, Beijing, China; 2 Graduate School, Peking Union Medical College, Chinese Academy of Medical Sciences, Beijing, China; 3 Centre for Infectious Diseases and Microbiology Laboratory Services, ICPMR– Pathology West, Westmead Hospital, University of Sydney, Darcy Road, Westmead, New South Wales, Australia; 4 Department of General Surgery, Peking Union Medical College Hospital, Chinese Academy of Medical Sciences, Beijing, China; The University of Hong Kong, HONG KONG

## Abstract

Accurate species identification of *Candida*, *Cryptococcus*, *Trichosporon* and other yeast pathogens is important for clinical management. In the present study, we developed and evaluated a yeast species identification scheme by determining the rDNA internal transcribed spacer (ITS) region length types (LTs) using a sequencer-based capillary gel electrophoresis (SCGE) approach. A total of 156 yeast isolates encompassing 32 species were first used to establish a reference SCGE ITS LT database. Evaluation of the ITS LT database was then performed on (i) a separate set of (n = 97) clinical isolates by SCGE, and (ii) 41 isolates of 41 additional yeast species from GenBank by *in silico* analysis. Of 156 isolates used to build the reference database, 41 ITS LTs were identified, which correctly identified 29 of the 32 (90.6%) species, with the exception of *Trichosporon asahii*, *Trichosporon japonicum* and *Trichosporon asteroides*. In addition, eight of the 32 species revealed different electropherograms and were subtyped into 2–3 different ITS LTs each. Of the 97 test isolates used to evaluate the ITS LT scheme, 96 (99.0%) were correctly identified to species level, with the remaining isolate having a novel ITS LT. Of the additional 41 isolates for *in silico* analysis, none was misidentified by the ITS LT database except for *Trichosporon mucoides* whose ITS LT profile was identical to that of *Trichosporon dermatis*. In conclusion, yeast identification by the present SCGE ITS LT assay is a fast, reproducible and accurate alternative for the identification of clinically important yeasts with the exception of *Trichosporon* species.

## Introduction

Fungi are a major cause of human disease especially in patients with immune compromise and serious underlying disease [1]. *Candida* species are the leading cause of fungemia, which in the USA, is ranked as the third most common cause of bloodstream infection in ICU patients with a crude mortality of 47.1% [2]. However, serious infections due to non-*Candida* yeasts, including *Cryptococcus*, *Trichosporon*, *Rhodotorula* and other previously rare species, are also increasingly [3–7]. Accurate and timely species identification is important for clinical management of patients because these yeast species have different antifungal susceptibilities and an increasing number e.g. *Candida glabrata*, *Candida krusei* and *Trichosporo*n species, are resistant or less susceptible to many antifungal agents [8, 9].

The limitations of conventional phenotypic methods for yeast identification are well known. Currently, rDNA gene complex sequencing targeted at the internal transcribed spacer (ITS) regions is considered as the “gold-standard” method for identifying yeast species [10–13]. However, this approach requires time for the sequencing process, is expensive and dependent on public sequence repositories which are not curated and contain errors [14]. Other molecular identification methods include reverse line blot (RLB) assays, rolling circle amplification (RCA) [9, 15, 16] and pyrosequencing [17, 18]. Proteomic approaches such as matrix-assisted laser desorption/ionization time of flight mass spectrometry (MALDI-TOF MS) also lends itself as an identification tool [19]. As a simple and less expensive alternative to ITS sequencing, another molecular identification method, sequencer-based capillary gel electrophoresis (SCGE) systems, has been reported to have good accuracy, reproducibility and inter-laboratory consistency, whilst retaining flexibility [20, 21].

In the present study, we applied SCGE to develop an identification scheme for the major pathogenic yeast species. We constructed an in-house database of rDNA ITS length types (LTs) of a large number (n = 156) and broad range of yeast species (n = 32) by SCGE. We then evaluated the performance of this LT library for its ability to identify a separate and unrelated set of 97 (nine species) clinical yeast isolates. To broaden the scope of the evaluation, and to examine for potential “cross-identifications” or species misidentifications, we also performed *in silico* analysis of the combined ITS1 and ITS sequences of 41 additional yeast species against the in-house database. The results of all SCGE-based identifications were compared against the definitive identification provide by a combined phenotypic and molecular approach established in our laboratory [9, 13, 22].

## Materials and Methods

### Ethics

The study was approved by the Human Research Ethics Committee of Peking Union Medical College Hospital (No. S-263). Written informed consent was obtained from patients for the use of the samples in research.

### Yeast isolates

(i) Database build set: A total of 156 isolates were used to construct the ITS LT database. These comprised five reference strains (*Candida parapsilosis* sensu stricto ATCC22019, *C*. *krusei* ATCC6258, *Candida guilliermondii* ATCC6260, *Candida albicans* ATCC90028 and *Trichosporon asahii* CBS2479) and 151 clinical isolates. The clinical isolates were part of the culture collection of the National China Hospital Invasive Fungal Surveillance Net (CHIF-NET) 2010 and 2011 [10, 13, 23] and encompassed 32 yeast species: *Candida* (21 species), *Cryptococcus* (four species), *Trichosporon* (four species) and other yeasts (three species) (see Table 1). All isolates were identified by MALDI-TOF MS (Vitek MS, bioMérieux, Marcy l’Etoile, France, database version IVD 2.0) according to the manufacturer’s instructions. Any isolates with unsatisfied identification confidence values (<99.9%), or with “no identification” results, were further identified by sequencing of the ITS region as described by Zhang *et al*. [13]. For non-*Trichosporon* isolates, this identification was taken as the definitive identification result. Definitive identification of *Trichosporon* species was provided by sequencing of the intergenic spacer 1 (IGS1) region [9].

**Table 1:** Summarization of ITS1 and ITS sizes called by SCGE and DNA sequencing for five reference strains and 151 clinical isolates used for establishment of the SCGE ITS length type (LT) database.

	ITS LT	No. of strains^b^	ITS1 region sizes called by (bp)		ITS region sizes called by (bp)		ITS GenBank	MALDI-TOF	
Species^a^			SCGE (Avg±SD^c^)	Sequencing	SCGE (Avg±SD^c^)	Sequencing	Accession no.	C/M/N^e^	Included in Vitek MS database^f^
**Reference strains**									
*Candida albicans*	cal-1	1/1	214.3±0.0^d^	218	532.9±0.0	536	KU052044	1/0/0	Yes
ATCC90028									
*Candida parapsilosis* sensu stricto	cpa-1	1/1	226.7±0.0^d^	229	517.5±0.0	520	KU052046	1/0/0	Yes
ATCC22019									
*Candida krusei*	ckr-1	1/1	176.4±0.1^d^	182	500.8±0.1	509	KU052057	1/0/0	Yes
ATCC6258									
*Candida guilliermondii*	cgu-1	1/1	245.0±0.1^d^	248	605.2±0.1	607	KU052058	1/0/0	Yes
ATCC6260									
*Trichosporon asahii*	tas-1	1/1	199.6±0.0^d^	203	538.4±0.0	541	KU052077	1/0/0	Yes
CBS2479									
**Clinical strains**									
***Candida* species**									
*Candida albicans*	cal-1	10/4	214.4±0.1	218	533.0±0.2	536	KU052044	10/0/0	Yes
	cal-2	11/2	215.4±0.1	219	534.0±0.2	537	KU052045	11/0/0	Yes
***Candida parapsilosis* species complex**									
*-Candida parapsilosis* sensu stricto	cpa-1	9/2	226.6±0.1	229	517.6±0.1	520	KU052046	9/0/0	Yes
*-Candida metapsilosis*	cme-1	8/2	232.9±0.1	236	528.1±0.2	531	KU052047	0/2/6	No
*-Candida orthopsilosis*	cor-1	2/2	213.0±0.1	220	508.2±0.0	511	KU052048	0/0/2	No
	cor-2	6/3	219.0±0.0	222	511.3±0.1	514	KU052049	0/1/5	No
*-Lodderomyces elongisporus*	lel-1	6/2	250.9±0.0	253	551.4±0.1	554	KU052050	0/0/6	No
*Candida tropicalis*	ctr-1	5/4	214.4±0.1	218	522.3±0.1	525	KU052051	5/0/0	Yes
	ctr-2	6/3	214.3±0.1	218	523.2±0.1	526	KU052052	6/0/0	Yes
***Candida glabrata* species complex**									
*-Candida glabrata* sensu stricto	cgl-1	3/2	480.0±0.1	482	880.2±0.1	881	KU052053	3/0/0	Yes
	cgl-2	7/2	481.3±0.3	483	881.3±0.3	882	KU052054	7/0/0	Yes
*-Candida nivariensis*	cni-1	1/1	359.2	361	756.7	758	KU052055	0/0/1	No
	cni-2	1/1	359.1	361	758.7	759	KU052056	0/0/1	No
*Candida krusei*	ckr-1	10/2	176.4±0.1	182	500.7±0.1	509	KU052057	10/0/0	Yes
*Candida guilliermondii*	cgu-1	10/3	245.1±0.1	248	605.4±0.1	607	KU052058	10/0/0	Yes
*Candida lusitaniae*	clu-1	5/4	142.9±0.1	148	378.9±0.1	383	KU052059	5/0/0	Yes
*Candida pelliculosa*	cpe-1	2/2	259.1±0.0	262	614.9±0.1	617	KU052060	2/0/0	Yes
	cpe-2	3/3	260.1±0.0	263	615.6±0.3	618	KU052061	3/0/0	Yes
***Candida haemulonii* species complex**									
*-Candida haemulonii*	cha-1	2/2	136.1±0.0	145	370.9±0.0	374	KU052062	2/0/0	Yes
*-Candida duobushaemulonii*	cdh-1	3/3	142.5±0.0	148	384.2±0.1	388	KU052063	0/3/0	No
*Candida norvegensis*	cno-1	1/1	180	187	483.7	491	KU052064	1/0/0	Yes
	cno-2	1/1	179.8	187	486.6	494	KU052065	1/0/0	Yes
	cno-3	1/1	180.9	188	485.2	493	KU052066	1/0/0	Yes
*Candida lipolytica*	cli-1	3/3	135.5±0.0	139	356.3±0.0	358	KU052067	3/0/0	Yes
*Candida fabianii*	cfa-1	2/2	266.8±0.0	270	619.5±0.3	622	KU052068	0/0/2	No
*Candida quercitrusa*	cqu-1	1/1	253.5	260	612.5	614	KU052069	0/0/1	No
*Candida intermedia*	cin-1	1/1	146	151	386	389	KU052070	1/0/0	Yes
*Candida catenulata*	cca-1	1/1	146.6	152	396.6	402	KU052071	1/0/0	Yes
*Candida rugosa*	cru-1	1/1	138.0±0.1^d^	143	389.8±0.0	398	KU052072	1/0/0	Yes
***Cryptococcus* species**									
***Cryptococcus neoformans* species complex**									
*Cryptococcus neoformans*	cne-1	8/4	197.5±0.1	201	552.5±0.2	555	KU052073	8/0/0	Yes
*Cryptococcus gattii*	cga-1	2/2	197.5±0.1	201	553.5±0.3	556	KU052074	0/1/1	No
*Cryptococcus laurentii*	cla-1	1/1	187.6	192	533	536	KU052075	1/0/0	Yes
*Cryptococcus curvatus*	ccu-1	1/1	188.6	192	526.2	528	KU052076	1/0/0	Yes
***Trichosporon* species**									
*Trichosporon asahii*	tas-1	6/2	199.5±0.1	203	538.4±0.1	541	KU052077	6/0/0	Yes
*Trichosporon japonicum*	tja-1	1/1	199.6±0.1^d^	203	538.2±0.1	541	KU052078	0/0/1	No
*Trichosporon asteroides*	tat-1	1/1	199.6±0.1^d^	203	538.3±0.1	541	KU052079	0/0/1	No
*Trichosporon dermatis*	tde-1	1/1	194.0±0.1^d^	197	526.4±0.1	528	KU052080	0/0/1	No
**Other species**									
*Rhodotorula mucilaginosa*	rmu-1	4/2	225.0±0.0	232	614.2±0.1	616	KU052081	4/0/0	Yes
*Kodamaea ohmeri*	koh-1	2/2	150.9±0.0	156	403.1±0.0	407	KU052082	2/0/0	Yes
	koh-2	1/1	151.9	157	404.6	408	KU052083	1/0/0	Yes
*Quambalaria cyanescens*	qcy-1	1/1	249.5	252	652.8	654	KU052084	0/0/1	No

Abbreviations: ITS, rDNA internal transcribed spacer region; LT, length type; SCGE, sequencer-based capillary gel electrophoresis; Avg±SD, average ± standard deviation.

^a^Refer to species identified by MALDI-TOF MS (Vitek MS, bioMérieux, Marcy l’Etoile, France) supplemented with ITS sequencing as proposed by Zhang *et al*. [13].

^b^Presented in no. of isolates of the LT/no. of isolates selected for sequencing.

^c^SD was calculated for LTs with ≥ 2 isolates.

^d^The five reference strains and one strain each of *C*. *rugosa*, *T*. *japonicum*, *T*. *asteroides* and *T*. *dermatis* were tested three times for evaluated the reproducibility of SCGE assay. And Avg±SD was calculated based on three times’ SCGE results.

^e^Refer to species identified by MALDI-TOF MS (Vitek MS, bioMérieux, Marcy l’Etoile, France). C, correct identification; M: misidentification; N: no identification

^f^Refer to species whether inclusion in the currently available Vitek MS v.2.0 database. Yes, species were included in the database; No, species were not included in the database.

(ii) Clinical evaluation set: A “test” set of 97 clinical isolates cultured from patients with invasive fungal diseases (IFDs) as part of routine care at the Peking Union Medical College Hospital (PUMCH) in 2013 (Table 2) were studied. Their identification was challenged the in-house built SCGE ITS LT database. None of the isolates used in this evaluation were employed to establish the database.

**Table 2:** The results of isolates collected from PUMCH in 2013 by SCGE.

Species^a^	SCGE ITS length type (LT)	No. of isolates
*Candida albicans*	cal-1	33
	cal-2	17
*Candida duobushaemulonii*	cdh-1	1
*Candida glabrata* sensu stricto	cgl-1	2
	cgl-2	5
*Candida guilliermondii*	cgu-1	2
*Candida krusei*	ckr-1	3
*Candida parapsilosis* sensu stricto	cpa-1	7
*Candida tropicalis*	ctr-1	6
	ctr-2	3
	ctr-3^b^	1
*Cryptococcus neoformans*	cne-1	15
*Cryptococcus gattii*	cga-1	2
**Total**	**-**	**97**

^a^Refer to species identified by MALDI-TOF MS (Vitek MS) supplemented with ITS sequencing as proposed by Zhang *et al*. [13].

^b^*C*. *tropicalis* LT ctr-3 was a new ITS LT (ITS1 length 214.5 bp, ITS length 521.1 bp called by SCGE) that was inconsistent with any existed *C*. *tropicalis* ITS LTs in the established SCGE ITS LT database (Table 1).

(iii) GenBank *in silico* analysis “test” set: In addition, 41 ITS sequences of 41 additional yeast species that were not involved in development of the ITS LT database were studied (Table 3). Their sequences were downloaded from the GenBank database for *in silico* analysis against the SCGE LT database to determine potential misidentifications.

**Table 3:** *In silico* analysis of 41 yeast species from GenBank not being involve in the ITS length type (LT) database.

Species	Strain ID no.	ITS1 region size (bp)	ITS region size (bp)	ITS GenBank accession no.
*Candida africana*	CBS 8781	233	551	AY342214
*Candida apicola*	CBS 4078	192	459	EU926481
*Candida bracarensis*	CBS 10154	385	805	GU199440
*Candida ciferrii*	CBS 5295	236	580	AY493435
*Candida colliculosa*	CBS133	373	797	KF300899
*Candida dubliniensis*	ATCC MYA-646	218	541	JQ070103
*Candida humicola*	NRRL Y-12944	198	533	FJ153213
*Candida inconspicua*	CBS 180	169	455	AB179767
*Candida kefyr*	CBS 712	309	721	EF568057
*Candida lambica*	ATCC 24750	165	452	AF218969
*Candida pararugosa*	ATCC 38774	164	415	AF421856
*Candida sake*	CBS 159	170	441	AJ549822
***Candida* species**				
*Candida sphaerica*	CBS 2105	307	722	AJ401711
*Candida utilis*	ATCC 22023	296	640	AF218990
*Candida valida*	CBS 189	166	484	DQ104712
*Candida zeylanoides*	CBS 619	272	626	DQ249202
***Cryptococcus* species**				
*Cryptococcus albidus*	ATCC 10666	234	621	JQ070095
*Cryptococcus uniguttulatus*	CBS 1730	212	624	AF444302
*Cryptococcus victoriae*	CBS 6550	213	520	AF444447
***Trichosporon* species**				
*Trichosporon cutaneum*	ATCC 28592	196	529	FJ943422
*Trichosporon dehoogii*	CBS 8686	196	528	AF444476
*Trichosporon domesticum*	CBS 8280	201	536	AF444414
*Trichosporon inkin*	CBS 5585	201	539	AF444420
*Trichosporon jirovecii*	CBS 6864	195	527	AF444437
*Trichosporon laibachii*	CBS 5790	196	531	AF444421
*Trichosporon moniliiforme*	ATCC46490	197	529	AF444480
*Trichosporon mucoides*	CBS 7625	197	528	AF444423
***Rhodotorula* species**				
*Rhodotorula glutinis*	CBS 20	231	606	AF444539
*Rhodotorula slooffiae*	CBS 7094	217	587	AF444552
*Rhodotorula nothofagi*	CBS 9091	227	609	AF444641
**Other species**				
*Bensingtonia yamatoana*	CBS 7243	249	623	AF444634
*Debaryomyces hansenii*	CBS 1795	278	639	EU816260
*Dipodascus capitatus*	CBS 197.35	175	325	AY788305
*Endomyces fibuliger*	CBS 329.83	296	654	AF218988
*Geotrichum candidum*	CBS 772.71	151	372	HE663404
*Hansenula anomala*	CBS 5759	263	619	DQ249196
*Malassezia furfur*	CBS 7019	289	820	AY743635
*Occultifur externus*	PYCC 4823	216	591	AF444642
*Pichia farinosa*	CBS 185	307	669	EF568067
*Rhodosporidium babjevae*	CBS 9071	232	607	AF444636
*Sakaguchia dacryoidea*	CBS 7999	225	625	AF444571

### ITS Duplex PCR

**Fig 1:**
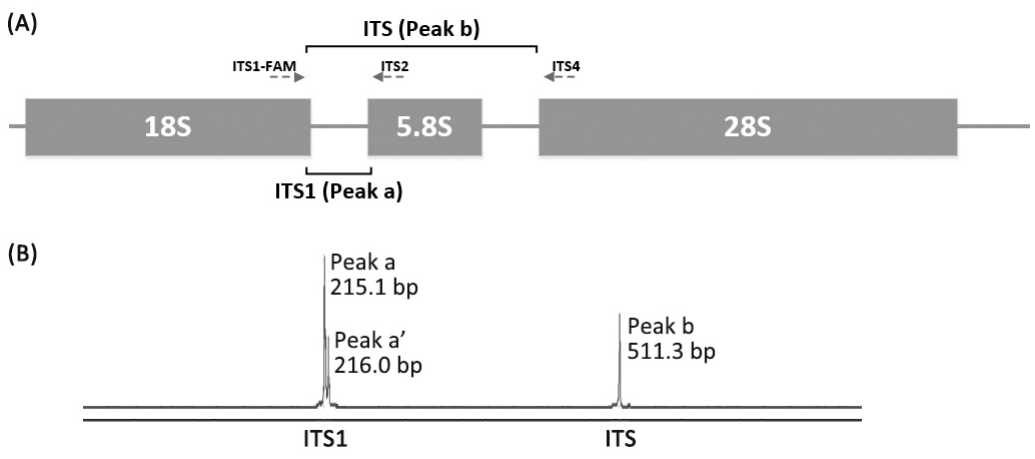
Principles for SCGE ITS length types (LTs) identification of yeast species. Fig 1A, structure of yeast ITS region and primers used in this study (showed in arrows with dashed lines). The duplex PCR using primers ITS1-FAM, ITS2 and ITS4 will theoretically generate two amplicons (for ITS1 and full-length ITS region, respectively). As the forward primer ITS1 was 5’-end FAM labeled, the amplicons can be observed by SCGE examination (peaks “a” and “b” in Fig 1B). B. an example of interpreting the SCGE results (strain 12HX414). Peaks “a” and “b” represent amplicons of ITS1 and full-length ITS region, respectively. As peak “a” and “a’” were separated by less than 1 bp, only peak “a” was counted (see Methods section).

For each isolate, both the ITS1 and full-length ITS regions were amplified by a duplex PCR using a unique forward primer ITS1-FAM (5’-TCCGTAGGTGAACCTGCGG-3’) and two reverse primers, ITS2 (5’-GCTGCGTTCTTCATCGATG-3’) and ITS4 (5’-TCCTCCGCTTATTGATATGC-3’), with the forward primer carboxyfluorescein (FAM)-labeled at the 5’end (Fig 1A). The duplex PCR was performed in a total volume of 25 μl: each reaction mixture contained 3 μl template DNA, 0.2 μl each of ITS1-FAM, ITS4 and 0.1 μl ITS2 primers (100 μM), 12.5 μl 2×*Taq* PCR StarMix (TransGen Biotech, Beijing, China), 9 μl molecular biology grade H_2_O (TransGen Biotech). The PCR parameters were: 95°C for 5 min, 30 cycles of 95°C for 1min, 52°C for 30 s, and 72°C for 1 min, followed by a final extension at 72°C for 10 min.

### Establishment of the ITS SCGE identification LT database

PCR fragment analysis was performed as before [20]. Generally, the ABI 3730xl DNA Analyzer (Applied Biosystems, Foster City, CA) was used employing a 96-capillary 50-cm POP-7 gel. The sample was added into the internal target (96-well plate), followed by vibration (500–1000 rpm for 1 min) and blending, denatured at 95°C for 5 min, rapidly cooled on the ice, and then centrifuged at 3700 rpm for 1 min. The supernatant was injected and the sample injection was carried out at 1.6 kV over 15 s with a total running time of 6,200 s at 15-kV run voltage. A 20- to 1,200-bp LIZ 1200 ladder (Chimerx, Madison, WI) was used as an internal marker for each sample.

The size of each peak was determined using GeneMarker software (Version 2.2.0, SoftGenetics, PA, USA). Double peaks were counted only if they were separated by more than 1 bp, otherwise only the highest peak was counted (Fig 1B). Between different strains, amplicons within ±0.5 bp differences was supposed to have the same length. LTs were defined by the combination of SCGE ITS1 and ITS amplicon lengths. Thereafter, the LTs and their matching “species identification” were recorded into an electronic database. The nomenclature of different ITS LTs was as follows: three lowercase Roman letters representing species, dash followed by an Arabic numeral representing potential length subtypes within each species. For example, “cor-2” represented species *Candida orthopsilosis*, ITS LT subtype 2 in the database.

One to four isolates of each LT were further selected to determine the physical lengths of the ITS1 and full-length ITS regions, by DNA sequencing, and to compare these with corresponding SCGE-derived lengths. The full-length ITS regions were amplified by primer pair ITS1/ITS4 as previously described [10] and sequenced from both directions. The physical lengths of the ITS1 and ITS regions (including primers) were analyzed by CLC Sequencing Viewer 7.5 (QIAGEN, Dusseldorf, Germany). The full-length ITS sequences were then used for phylogenetic analysis by the maximum-likelihood algorithm with 1000 bootstrap replication to ensure robustness using MEGA software (version 6.0, MEGA Inc., Englewood, NJ).

### SCGE reproducibility

SCGE was performed in triplicate on three separate occasions by three different technicians using fresh yeast subcultures for the five reference strains, and four isolates of rare *Candida* or non-*Candida* yeast species (*Candida rugosa*, *Trichosporon japonicum*, *Trichosporon asteroides* and *Trichosporon dermatis*).

### Clinical and *in silico* evaluation of the SCGE ITS LT database

Ninety-seven test isolates (seven *Candida* species, and two *Cryptococcus* species were used to evaluate the performance of the SCGE ITS LT database for yeast identification (Table 2). When a particular strain’s ITS LT exactly (with <0.5 bp difference) matched a recorded reference ITS LT in the database, it was designated as the corresponding species (Fig 1).

Because the ITS LT database did not cover all known species of yeasts, further, the ITS1 and ITS sequence length for 41 isolates of 41 additional yeast species, which were not included in the ITS LT database developed, were obtained from GenBank (Table 3). The ITS1 and ITS length of these isolates were *in silico* calculated, and queried against the developed ITS LT database to examine potential misidentifications.

## Results

### SCGE analysis of ITS1 and ITS amplicons

Duplex amplification of the ITS1 and ITS regions yielded positive PCR products in all 156 isolates for the ITS LT database development. Calling of the sizes of the ITS1 and ITS regions by SCGE are shown in Table 1 and Fig 1A. A total of 35 and 37 different amplicon lengths were obtained for the ITS1 and ITS region, respectively. The average amplicon lengths ranged from 135.5 bp to 481.3 bp for the ITS1 region and from 356.3 bp to 881.3 bp for the ITS region, with the standard deviation for different lengths ranging from 0–0.3 bp (Table 1). Of note, different physical lengths of the ITS1 and ITS regions obtained by DNA sequencing (Fig 1A) were clearly separated by SCGE, and amplicons with the same SCGE lengths had unique physical lengths (Table 1).

### Buildup of the SCGE ITS LT database

SCGE analysis of the five reference strains (four *Candida*, one *Trichosporon* species) revealed unique electropherograms, and established the premise of evaluating clinical strains.

**Fig 2:**
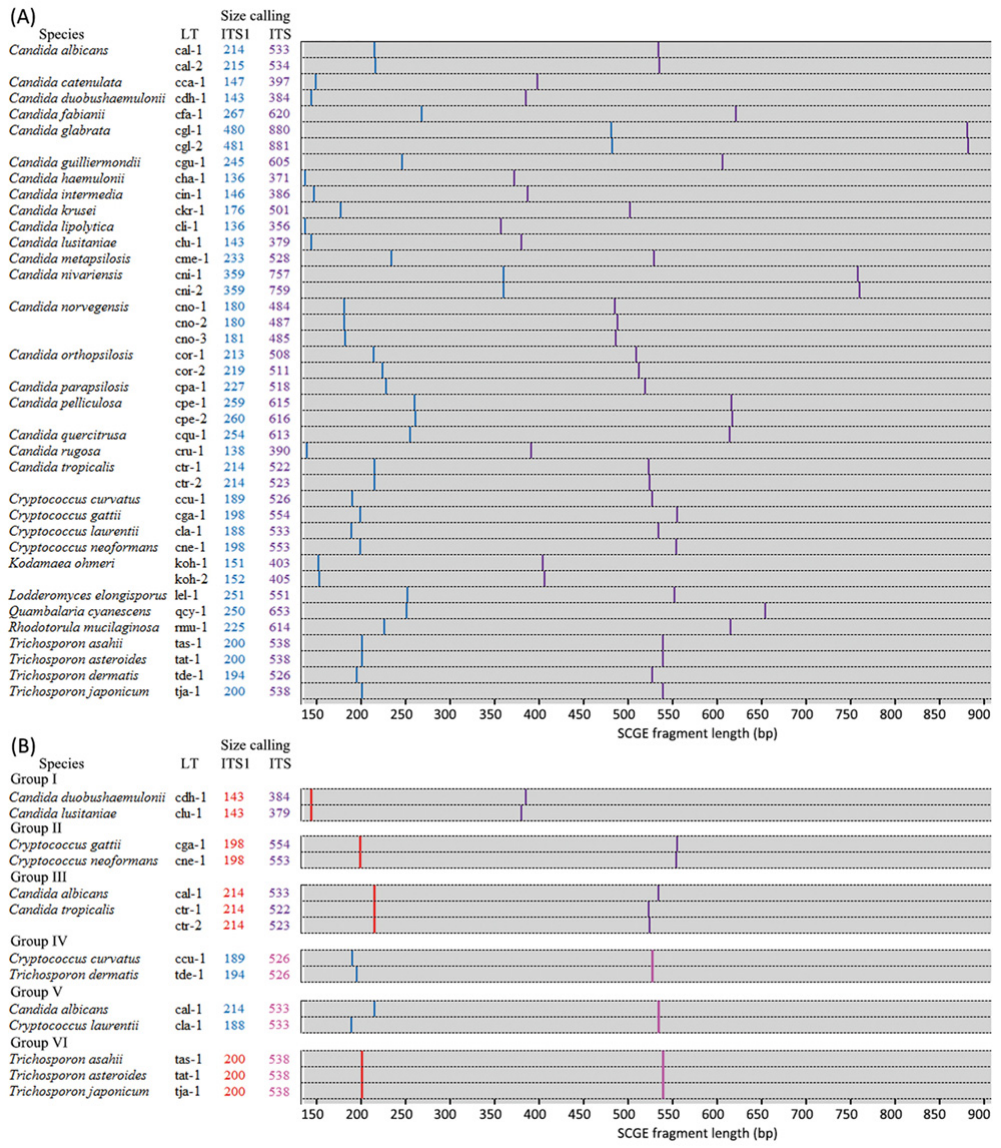
SCGE ITS length types (LTs) database established in the present study, including amplicon lengths (bp) of the ITS1 and ITS regions called by SCGE for each LT, and an *in silico* gel-view diagram. The amplicon sizes has been rounded up to the nearest whole number. Fig 2A, the whole database. Fig 2B, LTs of different species that had the same-length ITS1 or ITS region amplicons. In Fig 2A and 2B, gel-bands in blue or red represent amplicon of the ITS1 region, while gel-bands in purple or pink represent amplicon of the ITS region. Gel-bands in red or pink indicated the length of ITS1 or ITS amplicons were shared by different species.

Of 32 species represented amongst the 156 isolates, the ITS1 and full length ITS region directed SCGE assays distinguished 71.9% (23/32) and 78.1% (25/32) of the species, respectively. The ITS1 region lengths were identical for all *Candida duobushaemulonii* and *Candida lusitaniae* isolates (average length ± standard deviation 142.5±0.0 bp vs. 142.9±0.1 bp, Fig 2B Group I), between all *Cryptococcus neoformans* and *Cryptococcus gattii* isolates (197.5±0.1 bp vs. 197.5±0.1 bp, Fig 2B Group II), between all *Candida tropicalis* and the ITS LT cal-1 *C*. *albicans* isolates (214.4±0.1 bp vs. 214.3±0.1 vs. 214.4±0.1 bp, Fig 2B Group III), and between all *T*. *asahii*, *T*. *japonicum* and *T*. *asteroides* isolates (199.5±0.1 bp to 199.6±0.1 bp, Fig 2B Group VI) (Table 1). Meanwhile, the full-length ITS region was identical between all *Cryptococcus curvatus* and all *T*. *dermatis* isolates (526.2 bp vs. 526.4±0.1 bp, Fig 2B Group IV), *Cryptococcus laurentii* and the ITS LT cal-1 *C*. *albicans* isolates (533.0 bp vs. 533.0±0.2 bp, Fig 2B Group V), and *T*. *asahii*, *T*. *japonicum* and *T*. *asteroides* isolates (538.2±0.1 bp to 538.4±0.1 bp, Fig 2B Group VI) (Table 1).

**Fig 3:**
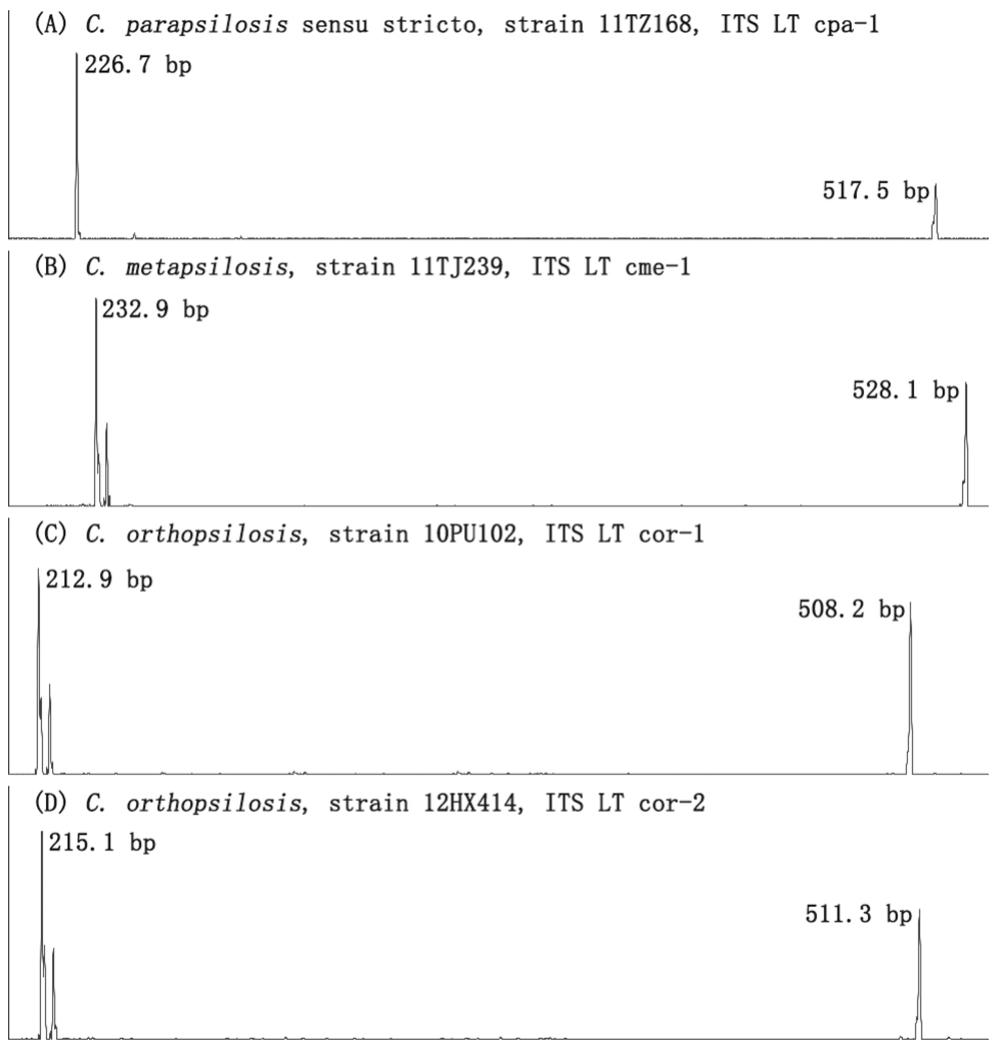
ITS length types (LTs) within *C*. *parapsilosis* species complex.

Based on the combination of ITS1 and ITS region lengths called by SCGE, 41 ITS LTs were identified, which was able to identify 94.9% (148/156) isolates of 90.6% (29/32) species used for SCGE ITS LT database development, only except for isolates of *T*. *asahii*, *T*. *japonicum* and *T*. *asteroides* (Fig 2B Group VI, Table 1). Of note, the ITS LTs were able to distinguish the following genetically closely related species within species complexes e.g. *C*. *parapsilosis* sensu stricto, *C*. *metapsilosis*, *C*. *orthopsilosis* and *L*. *elongisporus* within *C*. *parapsilosis* species complex (Fig 3), *Candida glabrata* sensu stricto and *Candida nivariensis* within *C*. *glabrata* species complex, *Candida haemulonii* and *C*. *duobushaemulonii* within *C*. *haemulonii* species complex, and *C*. *neoformans* and *C*. *gattii* isolates within *C*. *neoformans* species complex.

**Fig 4:**
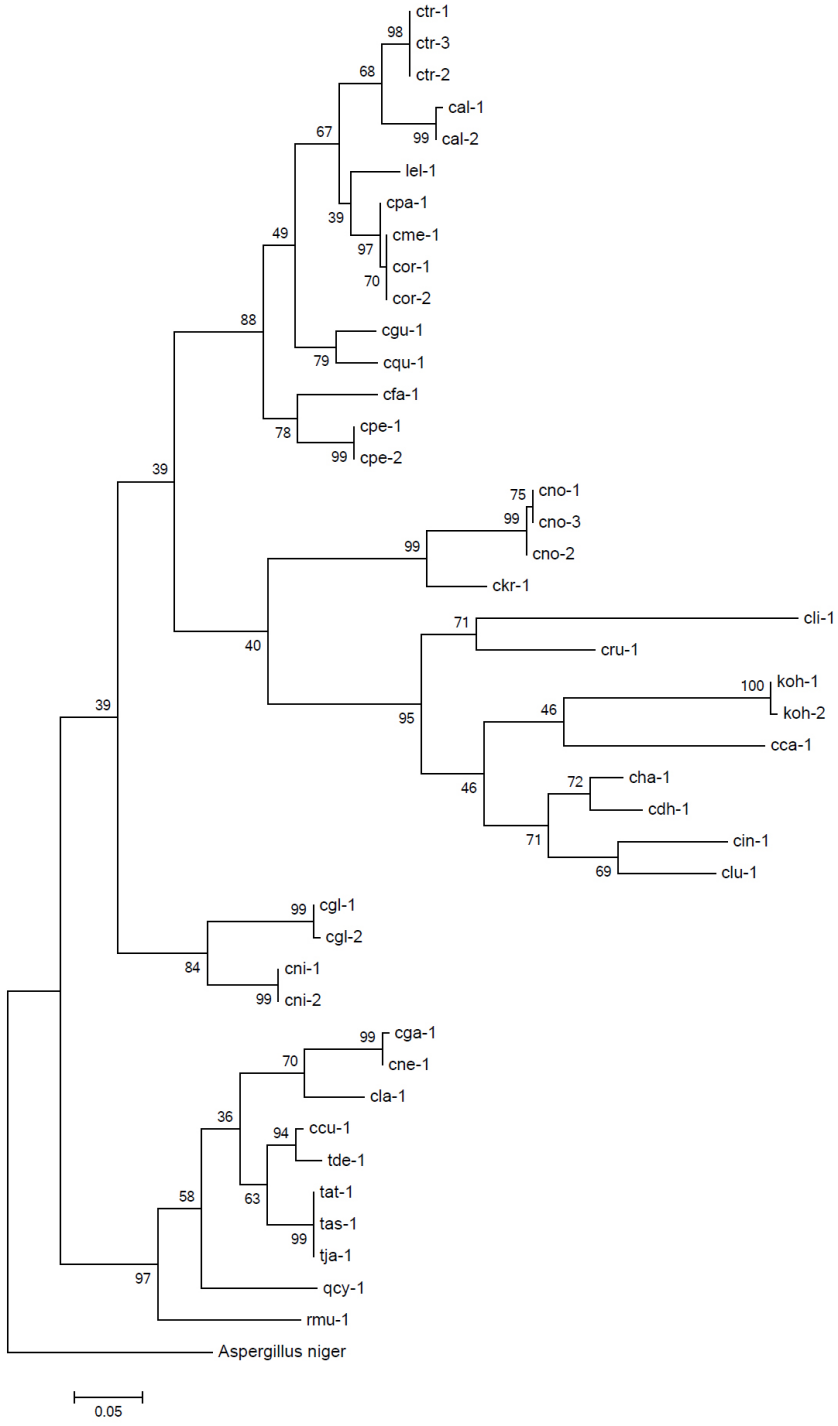
Phylogenetic tree based on the ITS sequences of 41 ITS LT isolates and ITS LT ctr-3 is shown and was conducted using the maximum-likelihood method, using *Aspergillus niger* ATCC 16888T as an outgroup.

Twenty-four of the 32 species studied had only one ITS LT, whilst eight species, including *C*. *albicans*, *C*. *orthopsilosis* (Fig 3C and 3D), *C*. *tropicalis*, *C*. *glabrata* sensu stricto, *C*. *nivariensis*, *Candida pelliculosa*, *Candida norvegensis* and *Kodamaea ohmeri* revealed 2–3 ITS LT subtypes (Table 1, Fig 1A). Dendrogram drawn by maximum-likelihood methods for full-length ITS sequences corresponding to 41 ITS LTs were shown in Fig 4. Different ITS LTs from the same or phylogenetically close-related yeast species, e.g. cal-1 and cal-2, cpa-1, cme-1, cor-1 and cor-2, cha-1 and cdh-1, cne-1 and cga-1, were clustered together.

### Reproducibility

Reproducibility of the SCGE technique was assessed by repeating the method on nine isolates (five reference strains and four clinical isolates) on three different occasions, and results are summarized in Table 1. For the same amplicon of each strain, the range of measured differences between different repeats was 0.0–0.1 bp.

### Clinical evaluation of the SCGE ITS LT database

The identification results by SCGE vs. the reference method (MALDI-TOF MS supplemented by DNA sequencing, see Methods) for the test set of 97 isolates is shown in Table 2. Using the ITS LT database, SCGE correctly identified 99.0% (96/97) isolates to species level, with 13 ITS LTs identified amongst the nine species studied. Only one isolate was not able to be identified—a putative “*C*. *tropicalis”* strain, which was not consistent and dissimilar to the ITS LTS of the two established *C*. *tropicalis* ITS LTs (i.e. ctr-1 and ctr-2, Table 1) in the database. ITS region sequence of the isolate was 99.6% identical to *C*. *tropicalis* type strain ATCC 750T (GenBank accession no. KJ651200), and phylogenetic analysis based on full-length ITS sequences clustered the isolate with ctr-1 and ctr-2 (Fig 4). In addition, the isolate was also identified as “*C*. *tropicalis*” by a variety of identification methods including API 20C AUX (bioMérieux), Vitek2 Compact YST (bioMérieux) and Vitek MS; hence, this isolate was assigned as *C*. *tropicalis*. A new ITS LT, ctr-3, was designated to this isolate, and ctr-3 was incorporated into the SCGE ITS LT database for future use.

### *In silico* evaluation of ITS LT database

The ITS1 and ITS region sizes of the additional 41 isolates of 41 yeast species were called, as showed in Table 3. Using the ITS LT database developed in the present study, only *Trichosporon mucoides* was “misidentified”, which had identical ITS1 and ITS lengths as *Trichosporon dermatis* ITS LT tde-1.

### Identification of yeast species by MALDI-TOF MS

Of 156 isolates used for SCGE-based ITS LT database, overall, 120 isolates (76.9%) were identified to species level by Vitek MS (bioMérieux), seven (4.5%) were misidentified, and 29 (18.6%) got “no identification” results (Table 1). Of the 32 yeast species studied, 20 species (62.5%) were included in the Vitek MS mass spectra database (database version IVD 2.0), whilst 12 species (37.5%) were absent from the database. All isolates of yeast species being included in the database (120 isolates) were correctly identified. However, amongst isolates of yeast species being absent from the database (36 isolates), 19.4% (seven isolates) were misidentified, and 80.6% (29 isolates) got “no identification” results.

## Discussion

The increasing spectrum and emergence of rare yeast pathogens continues to pose a challenge for microbiology laboratories to provide rapid and reliable identification [6, 19, 24, 25]. As a potential alternative molecular methods, here we have developed a SCGE-based approach for yeast identification and demonstrated that it can provide accurate and reproducible identification of major *Candida*, *Cryptococcus* and other rare yeast species with the exception of *Trichosporon* species.

By constructing an in-house database of SCGE patterns encompassing the electropherograms of 32 yeast species from six genera, we first established proof of principle that this technique was able to unambiguously identify and distinguish between the majority of the more commonly-encountered *Candida* and *Cryptococcus* species. Of note, both ITS1 and full length ITS SCGE amplicon results in combination were needed to make these distinctions allowing for correct identification of all isolates of these genera. Specifically, the ITS1-directed assay could not distinguish between certain *Candida* species including between *C*. *tropicalis* strains and one LT of *C*. *albicans*, both major *Candida* pathogens [10, 13, 23]. The inability of ITS1-diretced profiles on their own to differentiate between *C*. *neoformans* and *C*. *gattii* is consistent with the results of several ITS sequencing-based studies [26]. This reinforces the need for any method for fungal identification to be evaluated using more than one target, even within the same genetic locus (the ITS region in this case). Interestingly, the SCGE ITS LT assay was able to differentiate all genetically closed related species included in the study which cannot be differentiated by phenotypic methods [26–29]. This distinction is important for epidemiological studies and because there may be differences in antifungal susceptibilities between members within the complexes [28, 30].

Another key finding was that SCGE lends itself as a potential typing tool. It has been demonstrated that within the same yeast species, there might be different levels of intra-species ITS sequence diversity [11, 31]. Species with high genetic diversity are most frequently human commensals, and this finding could explain the existence of additional genetic adaptation within normal microbiota with older evolutionary origins [11]. As observed in this study, for eight of the species used to construct the database, subtypes based on their ITS LTs were identified, although from aspect of typing, SCGE was less discriminatory than multi-locus sequence typing [32, 33].

The results obtained with the “database build cohort” of isolates are supported by results obtained on the test isolates, albeit only representing nine species. Only one isolate could not be identified to species, and this isolate represented a novel subtype or ITS LT of *C*. *tropicalis*, again raising the possibility of using SCGE as a typing tool. The study of larger numbers of different yeast species in this context would be a clinical interest.

Comparing to the “gold standard” ITS sequencing methods, SCGE-based method was cheaper and with less turnaround time (Table 4). Although both methods relied on the expensive DNA analyzer equipment (e.g. ABI 3730xl, Applied Biosystems), clinical laboratories will benefit from widely available commercial DNA sequencing companies, who provide cost effective (~US$1.5 per SCGE sample analyzed) and timely (24 h) services in urban China (Table 4). MALDI-TOF MS, if available, is another powerful tool for yeast identification, which enables rapid and accurate identification of common clinically-important yeasts, but may be hindered by its equipment acquisition costs (Table 4) [13, 19]. Moreover, insufficiencies in yeast spectrum databases may affect its identification capacity in identifying close-related and rare yeast species (Table 1) [6, 13, 19, 25]. As shown in the present study, 37.5% of yeast isolates could not be correctly assigned to species level by Vitek MS system because the corresponding species were being absent from the mass spectra database.

**Table 4:** Comparison of SCGE, Vitek MS and ITS sequencing for identification of yeast species.

Assays	Turnaround time (hour)	Ease of preparation	Reagent costs per test (US$)	Set up cost of equipment
ITS sequencing	6^a^	complicated	4	expensive but can easily access commercial provider
SCGE	3^a^	complicated	1.5	expensive but can easily access commercial provider
Vitek MS	<1	easy	3	expensive

^a^For laboratory being absent from a DNA analyzer where samples need to be sent to commercial companies for tests, the turnaround time is supposed to be one working day in urban China.

In the broader sense, the usefulness of CGE-based assays for species identification of *Candida* has been reported previously by Monstein *et al*. and Mallus *et al*. [34, 35]. Both studies used the Seegene Seeplex PCR assay (Seegene Diagnostic, Seoul, South Korea) for nucleotide amplification, but CGE was carried out on two different platforms QIAxcel (Qiagen, Hilden, Germany) [35] and the MultiNA (Shimadzu Corp., Tokyo, Japan) [34], respectively. Our assay here differs as SCGE relies on fluorescent-labeled primers for PCR amplification whereas QIAxcel and MultiNA devices are adapted for analysis of conventional PCR products amplified by non-labeled primers requiring an additional step to identify nucleic acid with e.g. SYBR Green fluorescent stain. We chose the SCGE approach as the method has proved to be more sensitive in detecting low-intensity amplicons, more precise in size-calling, and has higher discriminatory power compared with the QIAxcel-based assay [36]. In addition, compared with previous CGE-focused *Candida* studies, that examined amplicons for less than 10 *Candida* species [34, 35], our study developed a relatively more comprehensive identification database, which comprised 21 *Candida* species, and 11 species of non-*Candida* yeasts e.g. *Cryptococcus*, *Trichosporon* and *Rhodotorula*. The isolates used for database development were from the CHIF-NET study, which was currently the largest nationwide multicenter surveillance program for IFDs in China, indicating its clinical relevance at least in the region.

The major limitation of the current SCGE ITS LT assay was its inability to identify *Trichosporon* species including *T*. *asahii*, *T*. *japonicum* and *T*. *asteroides*, *T*. *mucoides* and *T*. *dermatis*. It has been reported that the ITS and D1/D2 region sequences of above *Trichosporon* species were highly similar, with only few single nucleotide polymorphisms in nucleotide sequences but no difference in sequence-length [9, 37]. Therefore, the ITS region was not an appropriate target for identification of *Trichosporon* species. Instead the rDNA intergenic spacer (IGS) 1 region is most suitable for differentiating between phylogenetically close *Trichosporon* species because of its higher diversity [9, 37, 38]. Further work on IGS1 region for SCGE is warranted for study of this genus.

Another limitation of the study was that among the test 97 isolates, only seven *Candida* and two *Cryptococcus* species were analyzed. By studying 41 additional and including rare species of yeasts, by *in silico* analysis and by querying their identity against the ITS LT database, only *T*. *mucoides* was “misidentified” where the ITS LT profile was identical to that of *T*. *dermatis* (Table 3). This is encouraging and we plan to add to our in-house database the ITS LTS representative of more species so that it may be more widely applicable.

In summary, we have here established a database for SCGE-based identification for yeasts other than *Trichosporon* species, and the premise for performing a larger scale study to evaluate the identification capabilities of SCGE. To this end, DNA sequencing remains the “gold standard” identification method. However, the ITS SCGE assay described herein has shown promise to fulfill the function as a potential “reference” method since it is simple to use and adaptable for rapid identification. Moreover, it has the potential to detect mixed infections and to subtype species.
